# French Translation and Psychometric Evaluation of the Standardized Outcomes in Nephrology Life Participation Instrument Among Kidney Transplant Recipients

**DOI:** 10.1016/j.ekir.2026.106563

**Published:** 2026-04-21

**Authors:** Bénédicte Sautenet, Mathis Brier, Allison Jaure, Valentin Maisons, Magali Giral, Angela Ju, Yseulys Dubuy

**Affiliations:** 1 Department of Nephrology and Clinical Immunology, Tours Hospital, Tours, France; 2 Université de Tours, Nantes Université, INSERM UMR 1246, methodS in Patient-centered outcomes and HEalth ResEarch, SPHERE, Tours, France; 3 French Clinical Research Infrastructure Network-Cardiovascular and Renal Clinical Trialists, FCRIN INI-CRCT, Nancy, France; 4 Nantes Université, INSERM UMR 1229, Regenerative Medicine and Skeleton, RMeS, Nantes, France; 5 Institut des Hautes Etudes Ostéopathiques de Nantes (IdHEO), Laboratoire de Recherche en Ostéopathie (LRO), Nantes, France; 6 Sydney School of Public Health, The University of Sydney, Sydney, New South Wales, Australia; 7 Centre for Kidney Research, The Children's Hospital at Westmead, Westmead, New South Wales, Australia; 8 CHU Nantes, Nantes Université, Service de Néphrologique, Institut de Transplantation Uro-Néphrologique, Nantes, France; 9 Nantes Université, CHU Nantes, INSERM UMR 1064, Center for Research in Transplantation and Translational Immunology, CR2TI, Nantes, France; 10 Nantes Université, Université de Tours, INSERM UMR 1246, methodS in Patient-centered outcomes and HEalth ResEarch, SPHERE, Nantes, France

**Keywords:** kidney transplantation, life participation, patient-reported outcomes, questionnaire validation

## Abstract

**Introduction:**

The Standardized Outcomes in Nephrology – Life Participation (SONG-LP) instrument is a short patient-reported outcome measure designed to evaluate life participation among kidney transplant recipients (KTRs). Developed by the Standardized Outcomes in Nephrology (SONG) initiative, this tool has been validated in English, but no French version has been psychometrically evaluated.

**Methods:**

We translated the SONG-LP into French following a forward-backward translation process with linguistic validation. Between March and June 2023, 136 adult KTRs from 2 French university hospitals (Nantes and Tours) completed the SONG-LP and PROMIS-29 questionnaires during a follow-up transplant visit. We assessed acceptability, reliability (internal consistency and test-retest reproducibility), and construct validity (structural validity and hypothesis testing based on a confirmatory framework).

**Results:**

The SONG-LP score was computable for 99% of respondents, with minimal missing or “not applicable” responses. Ceiling effects were observed, particularly for the “work” and “social” items. Internal consistency was good (Cronbach’s α = 0.85) and test-retest reproducibility was acceptable (intraclass correlation coefficient ICC = 0.75). Results regarding construct validity were consistent with our *a priori* assumptions.

**Conclusion:**

The French version of the SONG-LP instrument is a valid and reliable tool for assessing life participation in KTRs.

## Graphical abstract

**Figure:**
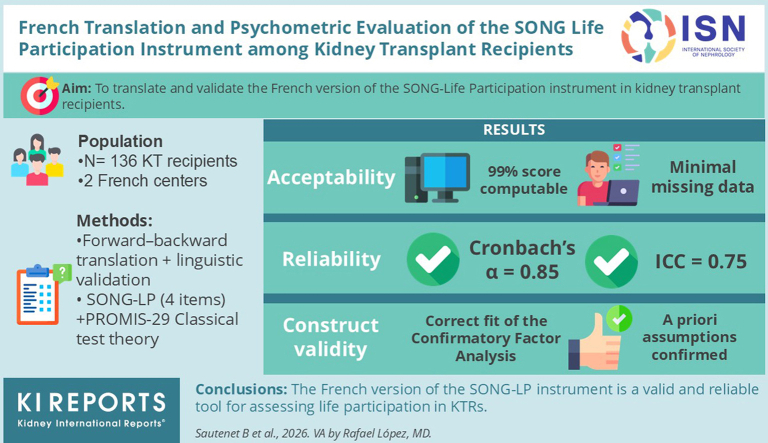

Kidney transplantation is the preferred treatment for patients with kidney failure requiring kidney replacement therapy, as it is associated with improved survival and health-related quality of life compared with dialysis.^1^, ^2^, ^3^, ^4^ However, kidney transplant recipients (KTRs) have a high risk of complications that can affect their ability to participate in daily life activities.^5^ The “ability to participate in meaningful activities of daily living, including work, study, and social and recreational activities”, termed as Life Participation (LP), has been identified as a critical outcome to be reported in all trials with KTRs by the Standardized Outcomes in Nephrology (SONG) initiative, which was based on the shared priorities of patients, caregivers, and health professionals.^6^

A recent systematic review highlighted the high heterogeneity of instruments used to assess LP in KTRs and the lack of data supporting their psychometric robustness for use in this population.^7^ The SONG initiative has developed a new instrument, the “SONG-LP instrument”, to provide a pragmatic and content-relevant questionnaire composed of positively worded items (i.e., items focusing on what respondents can do rather than what they cannot do), as highlighted during SONG workshops involving patients, caregivers, and health professionals.^8^ This instrument has been validated in English in adult KTRs.^6^

This study aims to translate the SONG-LP instrument into French and to assess the reliability and validity of this translation among French-speaking KTRs.

## Methods

### Participants

Between March and June 2023, consecutive KTRs attending their follow-up transplant visits at the French University Hospitals of Nantes and Tours were invited to participate in the study. Specifically, after explaining the study's rationale, clinicians asked patients whether they agreed to participate. Patients were eligible if they were ≥ 18 years, had received a kidney transplant > 1 month earlier, were living with a functioning kidney transplant, and were able to read and write in French and complete the questionnaire on their own. Patients with mental disorders were not eligible.

### Data Collection

After providing informed consent, KTRs were given a paper-based set of questionnaires to complete on their own at baseline. This set included the SONG-LP instrument and the PROMIS-29 questionnaire (French versions). Patients also had to self-report sociodemographic and clinical information (i.e., gender, age, marital situation, educational level, professional status, living or deceased donor, time since transplantation, cause of chronic kidney disease, and self-reported creatinine level). One week after the initial completion, participants from Nantes University Hospital were invited to complete the SONG-LP instrument again at home (retest, time 2). Ethical approval was obtained from the “*groupe nantais d’éthique dans le domaine de la santé*” from Nantes University Hospital (23-93-07-103).

### Measures

#### SONG-LP

The SONG-LP instrument is a self-reported questionnaire containing 4 items grouped into a single dimension. The 4 items assess the patient’s perceived ability to participate, over the past month, in the following: (i) leisure activities, (ii) family activities, (iii) work, and (iv) social activities. All items are scored on a 5-point response scale ranging from 1 = Never to 5 = Always. Each item also includes a “Not Applicable” response category.^6^ Item scores are recoded to range from 0 to 4, by subtracting 1 from all responses. An overall score for the LP instrument is calculated by averaging the patient’s recoded responses across all answered items (i.e., excluding items marked “Not Applicable”). The SONG-LP score ranges from 0 (indicating a low level of LP and a perceived inability to perform activities) to 4 (indicating a high level of LP and no perceived inability). The psychometric properties of the original questionnaire have been previously evaluated in English-speaking KTRs.^6^ Overall, the SONG-LP instrument appeared to be acceptable, internally consistent, and reliable. Correlations between the SONG-LP score and scores from other questionnaires were onsistent with the *a priori* hypotheses reported by Jaure *et al.*.^6^

**Figure 1:**
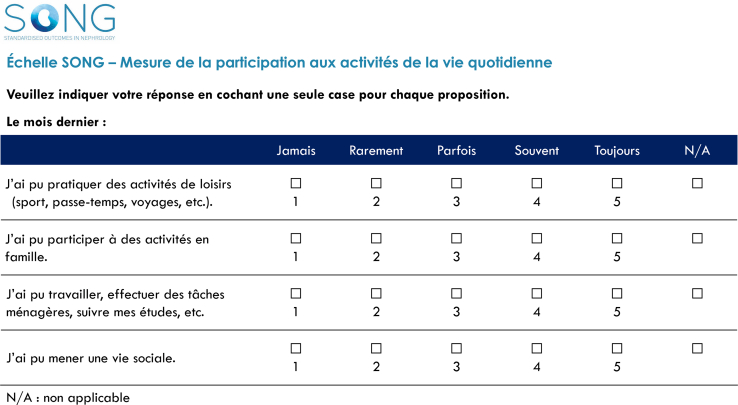
French translation for the SONG-LP instrument. SONG-LP, Standardized Outcomes in Nephrology – Life Participation.

To translate the SONG-LP instrument into French, we followed a forward-backward procedure. Specifically, the instrument was first translated into French by 4 translators (2 professional translators and 2 nephrologists) and then harmonized during a consensus meeting involving all translators. This unified translation was then back-translated by 2 independent professional translators. The final version was validated with a linguist to ensure conceptual equivalence through cultural and contextual adaptation. The French translation of the SONG-LP is provided in Figure 1.

#### PROMIS-29

The PROMIS-29 Profile version 2.0^9^ is a 29-item self-reported questionnaire assessing the following dimensions: physical function, anxiety, depression, fatigue, sleep disturbance, ability to participate in social roles and activities, pain interference, and pain intensity. The pain intensity dimension contains a single item with 11 response categories ranging from 0 to 10. All other dimensions contain 4 items scored on a 5-point response scale, with values ranging from 1 to 5 (the wording of the response categories varies depending on the item). For each of these multi-item dimensions, 2 scores can be computed as follows: (i) the raw score, computed as the sum of the responses to each item within the dimension, which ranges from 4 to 20, and (ii) the T-score, which derives from the raw score, computed following the PROMIS scoring manual. Regarding the physical and social domains, higher scores represent better functioning. For the other domains, higher scores reflect higher levels of anxiety, depression, fatigue, sleep disturbance, pain interference, and pain intensity.

### Data Analyses

#### Sample Characteristics

Sociodemographic and clinical characteristics were described using mean and SD for continuous variables and frequencies and percentages for categorical variables.

#### Psychometric Properties

##### Acceptability and Targeting

To ensure that the SONG-LP instrument was both acceptable and content relevant, we examined rates of missing values and “not applicable” responses, as well as potential floor and ceiling effects at the item level. Of note, item-level floor and ceiling effects were defined as a high proportion of respondents selecting the lowest (i.e., “Never”) or highest (i.e., “Always”) response category of each item, respectively. Such effects are worth investigating to determine whether a given item may be poorly targeted, either because it is related to an activity too demanding for the population assessed, resulting in a floor effect, or conversely too easy, resulting in a ceiling effect. At the score level, we calculated the percentage of KTRs for whom the SONG-LP score was computable (i.e., those who selected a response category between 1 and 5 for at least 1 item) and summarized the score distribution (range, mean, median, and SD). Floor and ceiling effects were also examined at the score level by computing the proportion of patients with an overall score of 0 (minimum) or 4 (maximum) respectively, to ensure that the instrument was capable of discriminating between respondents across the full range of life participation.

##### Structural Validity (Construct Validity)

We evaluated the structural validity of the SONG-LP by assessing the fit of a confirmatory factor analysis model, via the root mean square error of approximation (acceptable fit < 0.08), the comparative fit index (acceptable fit if > 0.95), and the standardized root mean square residual (acceptable fit if < 0.10).^10^

##### Association With Other Variables (Construct Validity)

The aim of these analyses was to test *a priori* assumptions regarding how the SONG-LP score correlates with other variables (i.e., gender, age, clinical data, and PROMIS-29 T-scores). Associations with gender and time since the transplantation (categorized into groups) were assessed using the Mann-Whitney and Kruskal-Wallis tests, respectively. Correlations with age, creatinine, and PROMIS-29 T-scores were assessed using the Spearman’s correlation coefficient $r_{S}$. Based on available literature and on clinical expertise, we expected:

• No correlation with age.^11^
• No association with gender.^11^
• A low negative correlation with self-reported creatinine level.^6^
• An association with time since transplantation. Specifically, clinicians hypothesized that LP could be impaired during the first year following the KT, although this result was not supported by the results of Jaure *et al.*^6^
• A high positive correlation (> 0.6) with the PROMIS-29 T-score “Ability to participate in social roles and activities,” since the SONG-LP instrument was adapted from PROMIS-Ability to Participate in Social Activities item bank.^6^ Of note, SONG-LP items have a positive wording, focusing on what respondents can do, whereas the PROMIS-29 “Ability to participate in social roles and activities” items have a negative wording (e.g., I have trouble doing …).
• Moderate correlations (between 0.3 to 0.6) with the PROMIS-29 T-scores “Depression,” “Fatigue,” and “Pain interference” (negative correlations), and “Physical function” (positive correlation).^12^
• Lower negative correlations with the PROMIS-29 “Anxiety” and “Sleep disturbance” scores.^12^

##### Reliability

The reliability of the SONG-LP instrument was evaluated by its internal consistency and its reproducibility between time points 1 (baseline) and 2 (retest). Internal consistency was assessed using Cronbach’s α coefficient (a value above 0.70 was considered as adequate). Reproducibility over time was assessed by comparing scores obtained at both time points (baseline and retest 1 week later). To determine whether scores were stable across these time points, we estimated an intraclass correlation coefficient (ICC) using a two-way analysis of variance with random effects. An ICC value above 0.75 was considered to indicate good reproducibility over time.^13^

### Software and Missing Data

Analyses were conducted using R statistical software (version 4.3.1, R Core Team) and Stata (version 18, StataCorp LLC, College Station, TX). Except for the acceptability and targeting analyses, missing data were addressed using personal-mean score imputation for participants with some missing items.

## Results

### Sample Characteristics

At time 1 (baseline), 139 individuals agreed to participate in the study. Of them, 136 participants met the inclusion criteria (n = 68 from each center: Nantes and Tours University Hospitals), as 3 patients were excluded because they were not KTRs. The mean age was 57.8 years (SD = 13.6), and 90 participants (66%) were men. The transplantation was from deceased donors for 103 (82%) participants, and 88 participants (66%) were transplanted less than 5 years ago. Other sociodemographic and clinical characteristics are presented in Table 1.

**Table 1:** *Sociodemographic and clinical characteristics of the sample at baseline (N= 136)*

	Mean (SD) or *n* (%)		Missing values
Age (yr)	57.8	(13.6)	*n* = 0
Sex			*n* = 0
Male	90	(66%)	
Female	46	(34%)	
Marital status			*n* = 0
Married/Partnered	96	(71%)	
Separated/Divorced	10	(7%)	
Widowed	5	(4%)	
Single	23	(17%)	
Other	2	(1%)	
Employment			*n =* 1
Full time	33	(24%)	
Part time	12	(9%)	
Casual	3	(2%)	
Unemployed	20	(15%)	
Retired	57	(42%)	
Student	0	(0%)	
Other	10	(7%)	
Transplant type			*n =* 8
Deceased	105	(82%)	
Living	23	(18%)	
Time since transplantation			*n* = 2
< 1 yr	45	(34%)	
1–5 yrs	44	(33%)	
6–10 yrs	18	(13%)	
> 10 yrs	27	(20%)	
Creatinine (μmol/l)	159.7	(113.6)	*n* = 40
Creatinine (mg/dl)	1.8	(1.3)	*n* = 40

n, frequency, %, percentage.

All these characteristics are self-reported by participants.

### Psychometric Properties

#### Acceptability and Targeting

Of the 136 participants, 9 (7%) did not respond or chose the response category “Not Applicable” for at least 1 item of the SONG-LP (see Supplementary Figure S1). At the item level, rates of missing values were low, ranging from <1% (leisure) to 3% (work). Similarly, “Not Applicable” response rates were about 1% for all items. Except for two participants whose questionnaires were completely missing or marked as “Not Applicable,” the SONG-LP score was computable for all other respondents (i.e., n = 134/136, 99%). Subsequent analyses will therefore focus on these 134 individuals. Of note, all SONG-LP items were missing for 1 patient, likely due to the corresponding page being inadvertently skipped, as the entire page (including all other information printed on it) was missing.

**Figure 2:**
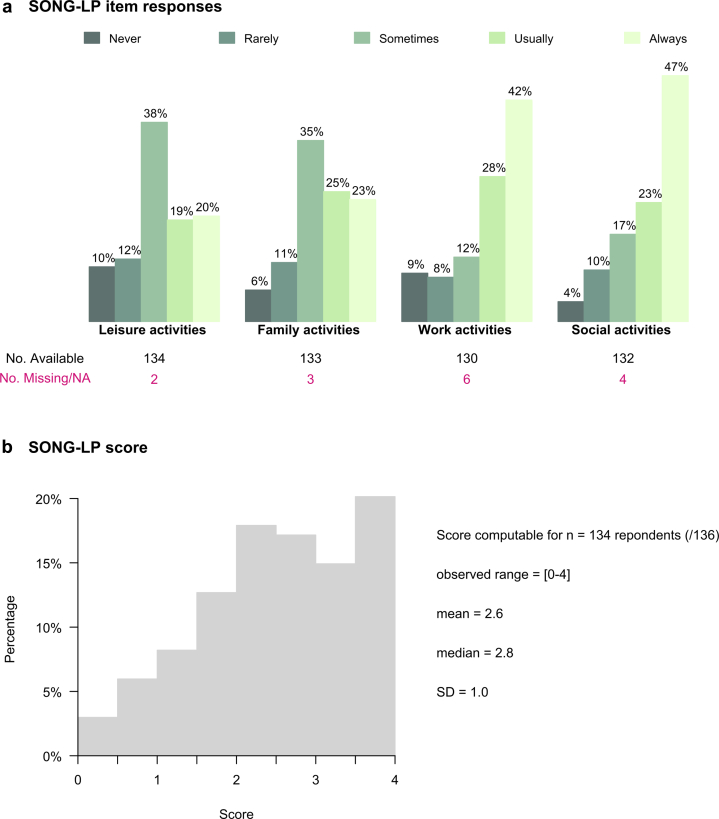
Acceptability and targeting: (a) Distribution of the SONG-LP item responses at baseline (bar chart). (b) Distribution of the SONG-LP score at baseline (histogram). SONG-LP, Standardized Outcomes in Nephrology – Life Participation, No. Available, Number of respondents who selected a scalable response category (i.e. between “Never” and “Always”), No. Missing/NA, Number of respondents who left the item empty (missing value) or selected the response category “Not Applicable”.

Ceiling effects were observed for the “work” and “social” items, with a large proportion of patients (> 30%) selecting the maximal response category “Always” for these items (42% and 47%, respectively), see Figure 2a. No floor effects were detected. Scores ranged from 0 to 4 (see Figure 2b). Of note, the above-mentioned item-level ceiling effects propagated to the score level, as 21 out of 134 respondents (16%) reached the maximal score (i.e., score = 4), indicating a score-level ceiling effect. In contrast, less than 1% of respondents obtained the minimal score (i.e., score = 0), suggesting no floor effect at the score level.

#### Structural Validity (Construct Validity)

**Figure 3:**
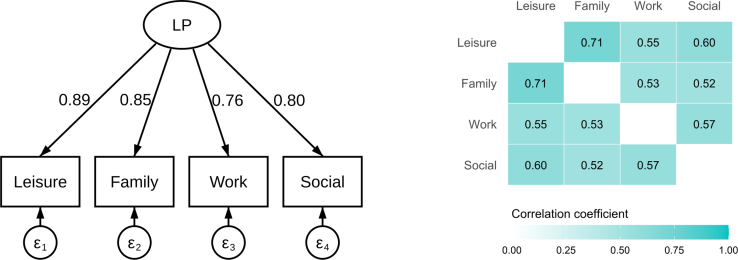
Structural validity (construct validity): Path diagrams of the confirmatory factor analysis (CFA) with standardized factor loadings and inter-item correlation heatmap (polychoric). LP, life participation.

The fit of the confirmatory factor analysis model was acceptable to good (root mean square error of approximation = 0.080, comparative fit index = 0.999, standardized root mean square residual = 0.033, diagonally weighted least squares estimation to account for ordinal data), and all standardized factor loadings were high (> 0.70, as shown in Figure 3), confirming the grouping of the 4 items into a single dimension.

#### Association with Other Variables (Construct Validity)

**Figure 4:**
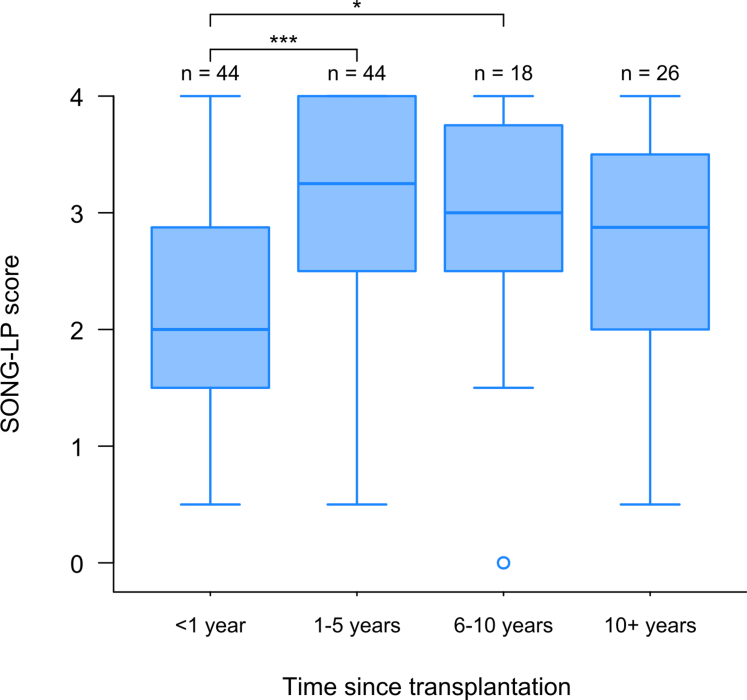
Association with other variables (construct validity): Distribution of the SONG-LP scores according to the time since transplantation (boxplot). Pairwise differences were investigated with the Dunn test, and *P*-values were adjusted using Bonferroni method. n: frequency, ∗ *P* < 0.05, ∗∗∗ *P* < 0.001, SONG-LP, Standardized Outcomes in Nephrology – Life Participation.

**Figure 5:**
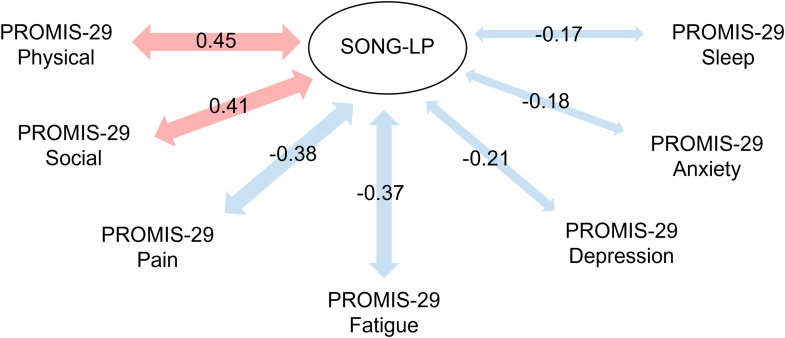
Association with other variables (construct validity): Spearman correlation coefficients between the SONG-LP score and the T-scores derived from the PROMIS-29 Profile. SONG-LP: Standardized Outcomes in Nephrology – Life Participation.

The SONG-LP score was not significantly associated with age ($r_{S}$ = -0.07, *P*-value = 0.392), gender (*P*-value = 0.133), or self-reported creatinine level ($r_{S}$ = -0.06, *P*-value = 0.597), see Supplementary Table S1. However, a significant association was evidenced with time since transplantation (*P* < 0.001). Specifically, patients who had undergone transplantation less than 1 year ago had lower score than other patients, see Figure 4. Finally, the pattern of associations between SONG-LP scores and PROMIS-29 T-scores was close to what we expected (Figure 5) as follows:

• A positive correlation was evidenced with the PROMIS-29 “Ability to participate in social roles and activities” T-score ($r_{S}$ = 0.41). However, the strength of this correlation was weaker than presumed (i.e., less than 0.6).
• As expected, moderate correlations were evidenced with the PROMIS-29 T-scores “Physical function” ($r_{S}$ = 0.45), “Fatigue” ($r_{S}$ = -0.37), “Pain interference” ($r_{S}$ = -0.38), and “Depression” ($r_{S}$ = -0.21, weaker than expected).
• Finally, in line with our assumptions, we found weaker correlations for the other PROMIS-29 T-scores (“Sleep disturbance” and “Anxiety”, $r_{S}$ = -0.17 and -0.18, respectively).

#### Reliability

**Figure 6:**
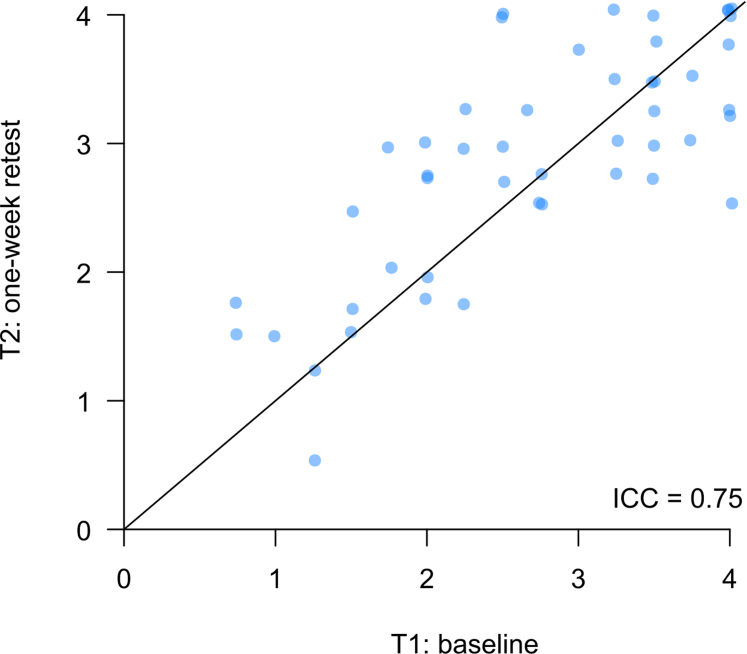
Test-retest reproducibility: Scatter plot of baseline (T1) versus one-week retest (T2) SONG-LP scores. ICC, intraclass correlation coefficient; SONG-LP, Standardized Outcomes in Nephrology – Life Participation.

The questionnaire showed good internal consistency, with a Cronbach’s α coefficient of 0.85. Among participants from Nantes, 48 (71%) participated in the retest 1 week later. The ICC was estimated at 0.75, indicating good reproducibility (Figure 6, Supplementary Figure S2).

## Discussion

This study aimed to translate and assess the psychometric properties of the French version of the SONG-LP instrument among KTRs. Our findings support the acceptability, reliability, and construct validity of the French translation.

The French version of the SONG-LP demonstrated excellent acceptability, with low rates of missing and “Not Applicable” responses, consistent with the pragmatic goal of the original instrument. Ceiling effects were observed at both the item and score levels, particularly for items assessing work and social activities. These effects may reflect the natural recovery process following transplantation, as patients typically experience improved functioning after the first year, corresponding to more than 60% of our patients.^4^ However, ceiling effects can limit the instrument's responsiveness in longitudinal studies. This emphasizes the importance of timing in the administration of patient-reported outcome measures and suggests that complementary instruments may be necessary when more granularity is required.^14^ Of note, such ceiling effects were also found by Jaure *et al.*^6^ in the testing of the English-language version of SONG-LP, affecting in their study all 4 items of the questionnaire and propagating to the score.

The structure with a single dimension seems to fit the data well, and the instrument demonstrated good internal consistency (Cronbach’s α = 0.85) and adequate test-retest reproducibility (ICC = 0.75), indicating stability of the measure over time in a stable patient population. These findings are in line with previous results obtained in English-speaking populations (Cronbach’s α = 0.87 and ICC = 0.62) and support the use of the French version in clinical and research settings.^6^

Our hypotheses regarding the associations between the SONG-LP score and sociodemographic, clinical, and PROMIS-29 measures were largely supported. As expected, and similar to the findings of Jaure *et al.*^6^ for the English-language version, no significant associations were observed between the SONG-LP score and age, gender, or creatinine level. Of note, creatinine levels were self-reported by participants rather than retrieved from medical records, which may have introduced inaccuracies and contributed to the substantial number of missing values observed for this variable; this is a limitation that should be considered when interpreting this particular finding. A significant association was found with time since transplantation, with lower scores among patients within the first-year following transplant. This finding, not reported in the original English-language validation study by Jaure *et al.*,^6^ may highlight a transient reduction in life participation during the postoperative recovery period and was already suspected.^15^

Correlations between the SONG-LP and PROMIS-29 dimensions were in line with our expectations, with moderate positive associations with physical function and ability to participate in social roles, and moderate negative associations with fatigue, pain interference, and depression. Of note, the correlation between the SONG-LP and PROMIS-29 “ability to participate in social roles and activities” dimension ($r_{S}$ = 0.41) was weaker than anticipated, despite both instruments theoretically measuring a similar construct. This finding may partly stem from differences in item phrasing between the 2 questionnaires (use of positive wording in the SONG-LP versus negative wording in the PROMIS-29). However, this attempt at explanation is not supported by the original English-language validation study by Jaure *et al.*,^6^ where a high correlation was evidenced between the 2 scores. Taken together, these observations suggest potential cross-cultural or cross-linguistic differences in how the items from the PROMIS-29 or SONG-LP are interpreted. Such differences may stem from semantic connotations (for instance, the interpretation of positive or negative phrasing may vary between French and English speakers), or from subtle shifts in item meaning introduced during the translation process of the SONG-LP to ensure natural phrasing in French (e.g., “I could do ∗*my*∗ leisure activities” was translated as “I could do leisure activities” potentially altering the specificity of the leisure activities conveyed in the original version).

Several limitations should be acknowledged. First, the sample size was moderate (n = 134), which may have limited the statistical precision of our analyses. Besides, recruitment was limited to 2 geographically close French regions, which may affect generalizability. Moreover, only patients from 1 center were invited for retest evaluation, potentially introducing center-specific bias. Future studies should aim to validate the French version in broader and more diverse French-speaking populations, including those from Canada, Belgium, Switzerland, and African countries. Finally, although the SONG-LP was designed to be short and pragmatic, it would also be interesting to collect information on perceived ease of completion, respondent burden, and willingness to complete the questionnaire.

To summarize, the SONG-LP instrument was designed as a pragmatic, brief tool to assess a core outcome prioritized by patients, caregivers, and clinicians which is the ability to participate in meaningful life activities. Its systematic use in all kidney transplantation trials is encouraged by the SONG initiative to improve patient-centeredness in transplant research. Our results support the use of the French version of the SONG-LP in this context, while highlighting the need for ongoing cross-cultural validation work to ensure valid comparisons across countries.

In conclusion, the French translation of the SONG-LP instrument demonstrates good psychometric properties among French KTRs. Its use in clinical trials and observational studies should be encouraged to capture patients’ perspectives on their life participation.

## Disclosure

All the authors declared no competing interests.

## Patient Consent

The study was conducted in accordance with the principles of the Declaration of Helsinki. Ethical approval was obtained from the Nantes University Hospital Ethics Committee (23-93-07-103). All participants provided written informed consent.

## Supplementary Material

Supplementary file (PDF) **Figure S1.** Response patterns with missing values and "not applicable" response categories at baseline. **Figure S2.** Test-retest reliability. **Table S1.** Association with other variables (construct validity).

## Footnotes

Supplementary file (PDF)
**Figure S1.** Response patterns with missing values and “not applicable” response categories at baseline. **Figure S2.** Test-retest reliability (reproducibility). **Table S1.** Association with other variables (construct validity).

## References

[1] Tonelli M., Wiebe N., Knoll G. (2011). Systematic review: kidney transplantation compared with dialysis in clinically relevant outcomes. Am J Transplant.

[2] Rose C., Gill J., Gill J.S. (2017). Association of kidney transplantation with survival in patients with long dialysis exposure. Clin J Am Soc Nephrol.

[3] Shi B., Ying T., Chadban S.J. (2023). Survival after kidney transplantation compared with ongoing dialysis for people over 70 years of age: a matched-pair analysis. Am J Transplant.

[4] de Boer S.E., Knobbe T.J., Kremer D. (2024). Kidney transplantation improves health-related quality of life in older recipients. Transpl Int.

[5] Knobbe T.J., Kremer D., Eisenga M.F. (2023). Sleep quality, fatigue, societal participation and health-related quality of life in kidney transplant recipients: a cross-sectional and longitudinal cohort study. Nephrol Dial Transplant.

[6] Jaure A., Vastani R.T., Teixeira-Pinto A. (2024). Validation of a core patient-reported outcome measure for life participation in kidney transplant recipients: the SONG life participation instrument. Kidney Int Rep.

[7] Ju A., Josephson M.A., Butt Z. (2019). Establishing a core outcome measure for life participation: a standardized outcomes in nephrology-kidney transplantation consensus workshop report. Transplantation.

[8] Tong A., Gill J., Budde K. (2017). Toward establishing core outcome domains for trials in kidney transplantation: report of the standardized outcomes in nephrology-kidney transplantation consensus workshops. Transplantation.

[9] Coste J., Rouquette A., Valderas J.M., Rose M., Leplège A. (2018). The French PROMIS-29. Psychometric validation and population reference values. Rev Epidemiol Sante Publique.

[10] Schermelleh-Engel K., Moosbrugger H., Müller H. (2003). Evaluating the fit of structural equation models: tests of significance and descriptive goodness-of-fit measures. https://www.psycharchives.org/en/item/1a8dea48-0285-4dac-a612-9dc0ff2532f6.

[11] Tang E., Ekundayo O., Peipert J.D. (2019). Validation of the Patient-Reported Outcomes Measurement Information System (PROMIS)-57 and −29 item short forms among kidney transplant recipients. Qual Life Res.

[12] Hartmann C., Fischer F., Klapproth C.P., Röhle R., Rose M., Karsten M.M. (2023). PROMIS-29 and EORTC QLQ-C30: an empirical investigation towards a common conception of health. Qual Life Res.

[13] Koo T.K., Li M.Y. (2016). A guideline of selecting and reporting intraclass correlation coefficients for reliability research. J Chiropr Med.

[14] Al Sayah F., Jin X., Johnson J.A. (2021). Selection of patient-reported outcome measures (PROMs) for use in health systems. J Patient-Rep Outcomes.

[15] De Beir J., De Baets S., Vandecruys M. (2024). Challenges in posttransplantation care for kidney transplant recipients: a qualitative study highlighting gaps in psychological, social and exercise support. J Ren Care.

