# Supplementary material for: French Translation and Psychometric Evaluation of the Standardized Outcomes in Nephrology Life Participation Instrument Among Kidney Transplant Recipients
Source: Kidney Int Rep. 2026 Apr 21;11(7):106563. doi: 10.1016/j.ekir.2026.106563 (PMC13234726; doi:10.1016/j.ekir.2026.106563)

ESM: ELECTRONIC SUPPLEMENTARY MATERIALS

French translation and psychometric evaluation of the SONG Life Participation instrument among kidney transplant recipients

Bénédicte Sautenet*, Mathis Brier*, Allison Jaure, Valentin Maisons, Magali Giral, Angela Ju, Yseulys Dubuy

shared first authorship

Figure S.1: Response patterns with missing values and “not applicable” response categories at baseline

Notes:

- *Each line represents a response pattern where missing values and/or “not applicable” response categories were observed.*
- *Grey boxes indicate items for which a response between 'Never' and 'Always' was selected by the patients. Pink boxes represent items with missing values, and violet boxes represent items marked as “not applicable”.*

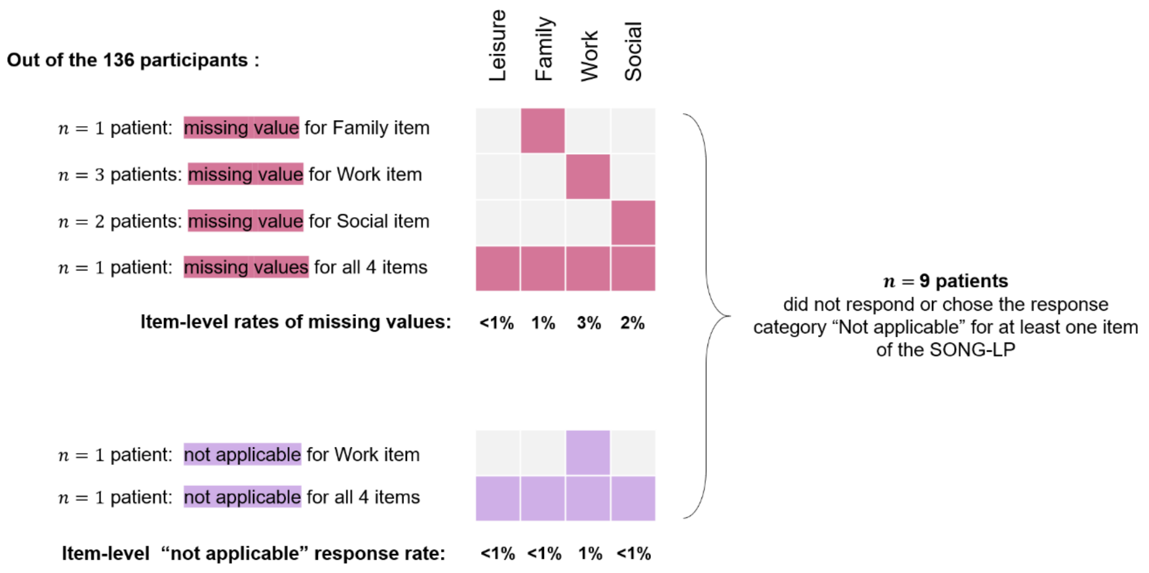

Of note, all SONG-LP items were missing for one patient, likely due to the corresponding page being inadvertently skipped, as the entire page (including all other information printed on it) was missing.

Table S.1: [Association with other variables (construct validity)]

A. SONG-LP score according to gender and time since transplantation

B. Correlations between the SONG-LP score and age, self-reported creatinine, and PROMIS-29 T-scores

**(A) SONG-LP score according to gender and time since transplantation**	*Median [Q1, Q3]*
**Gender**	p-value = 0.133
Male (n = 88)	2.8 [2.0, 3.5]
Female (n = 46)	2.5 [1.5, 3.2]
**Time since transplantation**	p-value < 0.001
< 1 year	2.0 [1.5, 2.8]
1-5 years	3.2 [2.5, 4.0]
6-10 years (n = 18)	3.0 [2.5, 3.7]
10+ years (n = 26)	2.9 [2.1, 3.5]

**(B) Spearman's correlation coefficients between the SONG-LP score and age, self-reported creatinine, and PROMIS-29 T-scores**	r_S (95% CI)
**Age**	-0.07 (-0.24, 0.10)
**Creatinine**	-0.06 (-0.25, 0.15)
**PROMIS-29 Physical function**	0.45 (0.31, 0.58)
**PROMIS-29 Ability to participate in social roles and activities**	0.41 (0.26, 0.54)
**PROMIS-29 Pain interference**	-0.38 (-0.52, -0.22)
**PROMIS-29 Fatigue**	-0.37 (-0.51, -0.21)
**PROMIS-29 Depression**	-0.21 (-0.37, -0.04)
**PROMIS-29 Anxiety**	-0.18 (-0.34, -0.01)
**PROMIS-29 Sleep**	-0.17 (-0.33, 0.00)

Notes:
Q1, Q3: first and third quartile, respectively
95% CI: 95% confidence interval

Figure S.2: [Test-retest reliability]

Bland-Altman plot of baseline (T1) and one-week retest (T2) SONG-LP scores. The solid line represents the mean bias and the dashed lines represent the 95% limits of agreement.

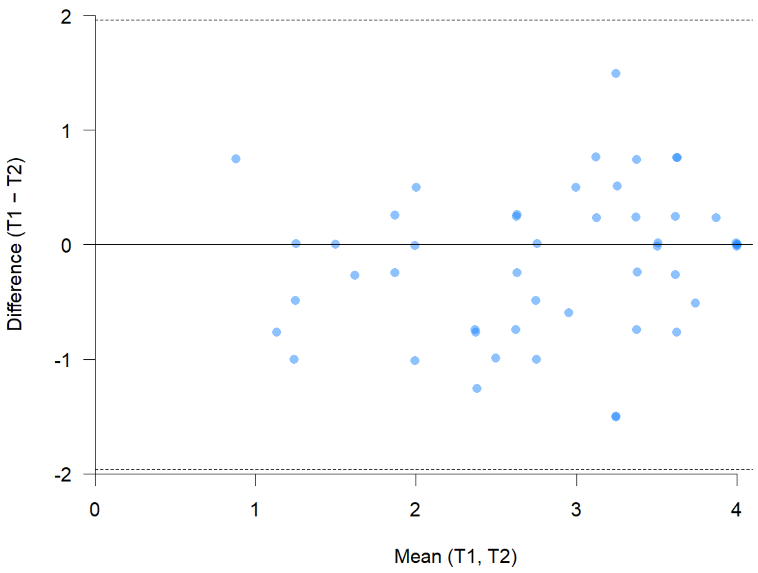

Supplement: Supplementary file (PDF) — Figure S1. Response patterns with missing values and "not applicable" response categories at baseline. Figure S2. Test-retest reliability. Table S1. Association with other variables (construct validity). [file mmc1.pdf]
